# Annual changes in grip strength and skeletal muscle mass in chronic liver disease: observational study

**DOI:** 10.1038/s41598-023-28528-w

**Published:** 2023-01-30

**Authors:** Kei Endo, Keisuke Kakisaka, Hidekatsu Kuroda, Akio Miyasaka, Yasuhiro Takikawa, Takayuki Matsumoto

**Affiliations:** Division of Gastroenterology and Hepatology, Department of Internal Medicine, Iwate Medical University School of Medicine, Idaidori 2-1-1, Yahaba, Iwate 028-3695 Japan

**Keywords:** Hepatology, Nutrition, Geriatrics

## Abstract

Sarcopenia is a common complication in patients with chronic liver disease (CLD); however, the progression of sarcopenia over the course of CLD is unclear. The present study therefore determined the natural course of the progression of sarcopenia in patients with CLD and the effect of liver cirrhosis (LC) on this progression. This observational study analyzed patients with chronic hepatitis (CH) (n = 536) and LC (n = 320) who underwent evaluations of the grip strength and skeletal muscle mass of the arms, trunk, and legs for sarcopenia between 2016 and 2021. A bioelectrical impedance analysis was used to evaluate skeletal muscle mass. The annual rate of change (%/year) in two tests were compared between patients with CH and LC. The annual rates of change in grip strength and skeletal muscle of arms, trunk, and legs of patients with CH and LC were − 0.84% vs. − 2.93%, − 0.54% vs. − 1.71%, − 0.43% vs. − 1.02%, and − 0.76% vs. − 1.70% for men and − 0.12% vs. − 1.71%, − 0.66% vs. − 1.71%, − 0.49% vs. − 1.31%, and − 0.76% vs. − 1.54% for women, respectively. The progression of sarcopenia was greater in LC patients than in CH patients and that the decrease in grip strength was most prominent in the progression of sarcopenia in patients with LC.

## Introduction

Sarcopenia is defined as the progressive and systemic loss of skeletal muscle mass and strength or physical performance. It is adversely associate with the quality of life (QOL) and clinical outcomes^1–4^. Its clinical importance has been highlighted in the field of chronic liver disease (CLD) as well as in various areas of clinical practice^5–8^. Liver cirrhosis (LC) is a representative cause of secondary sarcopenia as it is caused by something other than ageing (e.g., chronic inflammatory disease)^2^.

The diagnosis of sarcopenia is based on the loss of muscle strength and skeletal muscle mass or physical performance^2–4,9^. Grip strength is used as an indicator of muscle strength, and computed tomography (CT), a bioelectrical impedance analysis (BIA), and dual-energy X-ray absorptiometry (DEXA) are mainly used to evaluate skeletal muscle mass. A BIA and DEXA are the modalities most commonly used to diagnose primary sarcopenia due to aging, while CT is often used to diagnose secondary sarcopenia caused by liver disease or cancer, as it has the advantage of being able to examine the primary disease at the same time^2–4,9–13^. CT measures the skeletal muscle mass of the trunk (third lumbar vertebra level), and a BIA and DEXA measure the skeletal muscle mass of the extremities. Therefore, the measurement sites differ among the established methods. Muscle strength assessment by grip strength and skeletal muscle mass assessment by the BIA method have been adopted and are widely used as criteria for the Asian Working Group for Sarcopenia (AWGS)^3^. Furthermore, the BIA method is suitable for repeated evaluations because there is no radiation exposure, and each body part can be measured separately. The age-related loss of skeletal muscle mass and strength has also been reported to vary by body part and sex^14–17^. However, how sarcopenia progresses in patients with CLD is unclear. It is important to understand the progressive form of sarcopenia because it will help to determine where resistance training should be concentrated during treatment.

The present study determined the natural course of sarcopenia progression in patients with CLD and the effect of cirrhosis on sarcopenia progression by assessing the chronological changes in skeletal muscle mass and grip strength.

## Results

### Baseline characteristics

**Figure 1:**
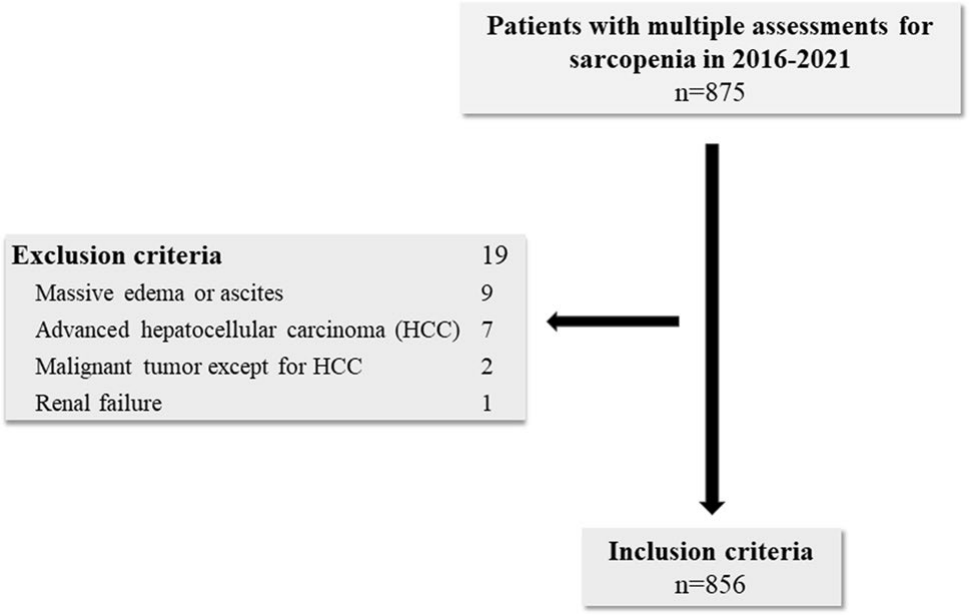
Flow chart of patient selection.

From 2016 to 2021, 875 patients were evaluated for sarcopenia using the BIA method at our institution at least twice. Based on the inclusion and exclusion criteria, a total of 19 patients were excluded. The details of patient selection are shown in Fig. 1.

Eight hundred and fifty-six patients were enrolled in this study. The clinical characteristics of the patients are shown in Table 1. There were 469 men and 387 women with a median age of 66 and 68 years old, respectively. LC, HCC, and diabetes mellitus were significantly more common in men than in women. The skeletal muscle mass in each region and grip strength were significantly higher in men than in women, while the total fat mass was significantly higher in women than in men.

**Table 1:** Baseline characteristics.

	Men n = 469	Women n = 387	*P*
Age (years)	65.3 (10.8)	67.2 (10.8)	0.01
Liver cirrhosis	208 (44.3)	112 (28.9)	< 0.01
Etiology			
HBV	78 (16.6)	31 (8.0)	
HCV	207 (44.2)	184 (47.6)	
Alcohol	93 (19.8)	10 (2.6)	< 0.01
NAFLD	76 (16.2)	105 (27.1)	
Others	15 (3.2)	57 (14.7)	
Modified ALBI grade			0.02
1	368 (78.5)	332 (85.8)	
2a	47 (10.0)	27 (7.0)	
2b/3	54 (11.5)	28 (7.2)	
Hepatocellular carcinoma	100 (21.3)	29 (7.5)	< 0.01
Diabetes mellitus	175 (37.3)	117 (30.2)	0.03
Body mass index (kg/m^2^)	25.3 (4.4)	25.3 (4.8)	0.99
Skeletal muscle index (kg/m^2^)	7.74 (0.96)	6.36 (0.90)	< 0.01
Skeletal muscle index of arms (kg/m^2^)	2.11 (0.33)	1.62 (0.31)	< 0.01
Skeletal muscle index of trunk (kg/m^2^)	8.47 (0.95)	7.33 (0.90)	< 0.01
Skeletal muscle index of legs (kg/m^2^)	5.64 (0.68)	4.73 (0.64)	< 0.01
Grip strength (kg)	36.4 (7.3)	21.9 (5.0)	< 0.01
Total fat mass (kg)	19.8 (9.5)	22.1 (9.2)	< 0.01
Sarcopenia	38 (8.1)	35 (9.0)	0.63
Low skeletal muscle index	89 (19.0)	86 (22.2)	0.27
Low grip strength	60 (12.8)	85 (22.0)	< 0.01

Values are presented as the mean (SD) or number (%).

ALBI, albumin-bilirubin; HBV, hepatitis B virus; HCV, hepatitis C virus; NAFLD, nonalcoholic fatty liver disease.

A low SMI and low grip strength were found in 89 (19.0%) and 60 (12.8%) men and 86 (22.2%) and 85 (22.0%) women, respectively. Ultimately, 38 (8.1%) men and 35 (9.0%) women were diagnosed with sarcopenia.

### The comparison of baseline characteristics between CH and LC

The clinical characteristics of CH and LC patients are shown in Table 2. For both men and women, LC patients were significantly older than CH patients (*P* < 0.01, *P* < 0.01). In men, both grip strength and skeletal muscle mass in each region were significantly lower in LC patients than in CH patients (*P* < 0.01), while there was no significant difference in total fat mass (*P* = 0.09). In women, there were no significant differences in total fat mass or skeletal muscle mass in each region, but LC patients had a significantly lower grip strength than CH patients (*P* < 0.01). Consequently, the prevalence of sarcopenia was significantly higher in LC patients than in CH patients of both sexes (*P* < 0.01).

**Table 2:** The comparison of patients with chronic hepatitis and liver cirrhosis.

	Men			Women		
	Chronic hepatitis (n = 261)	Liver cirrhosis (n = 208)	*P*	Chronic hepatitis (n = 275)	Liver cirrhosis (n = 112)	*P*
Age (years)	62.8 (10.8)	68.5 (10.0)	< 0.01	65.7 (10.8)	71.0 (9.9)	< 0.01
Etiology			< 0.01			< 0.01
HBV	57 (21.8)	21 (10.1)		23 (8.4)	8 (7.2)	
HCV	126 (48.3)	81 (38.9)		128 (46.5)	56 (50.0)	
Alcohol	19 (7.3)	74 (35.6)		1 (0.4)	9 (8.0)	
NAFLD	54 (20.7)	22 (10.6)		79 (28.7)	26 (23.2)	
Others	5 (1.9)	10 (4.8)		44 (16.0)	13 (11.6)	
Hepatocellular carcinoma	13 (5.0)	87 (41.8)	< 0.01	3 (1.1)	26 (23.2)	< 0.01
Diabetes mellitus	80 (30.7)	95 (45.7)	< 0.01	68 (24.7)	49 (43.8)	< 0.01
Body mass index (kg/m^2^)	25.8 (4.6)	24.8 (4.1)	0.02	25.4 (4.7)	25.3 (5.0)	0.95
Skeletal muscle index (kg/m^2^)	7.88 (0.88)	7.57 (1.04)	< 0.01	6.38 (0.86)	6.30 (0.99)	0.43
Skeletal muscle index of arms (kg/m^2^)	2.16 (0.32)	2.05 (0.34)	< 0.01	1.63 (0.29)	1.61 (0.34)	0.61
Skeletal muscle index of trunk (kg/m^2^)	8.62 (0.92)	8.29 (0.95)	< 0.01	7.36 (0.85)	7.26 (1.00)	0.32
Skeletal muscle index of legs (kg/m^2^)	5.72 (0.60)	5.52 (0.75)	< 0.01	4.75 (0.61)	4.69 (0.71)	0.40
Grip strength (kg)	38.2 (6.8)	34.0 (7.4)	< 0.01	22.5 (4.8)	20.3 (5.1)	< 0.01
Total fat mass (kg)	20.5 (10.1)	18.9 (8.6)	0.08	22.3 (9.2)	21.5 (9.2)	0.43
Sarcopenia	11 (4.2)	27 (13.0)	< 0.01	16 (5.8)	19 (17.0)	< 0.01
Low skeletal muscle index	36 (13.8)	53 (25.5)	< 0.01	55 (20.0)	31 (27.7)	0.11
Low grip strength	16 (6.1)	44 (21.2)	< 0.01	46 (16.7)	39 (34.8)	< 0.01

Values are presented as the mean (SD) or number (%).

HBV, hepatitis B virus; HCV, hepatitis C virus; NAFLD, nonalcoholic fatty liver disease.

### Annual rate of change in sarcopenia-relevant factors

A total of 601 patients were re-assessed for sarcopenia during the subsequent 1–2 years after the initial assessment. The median interval was 1.53 (1.01–5.26) years.

**Figure 2:**
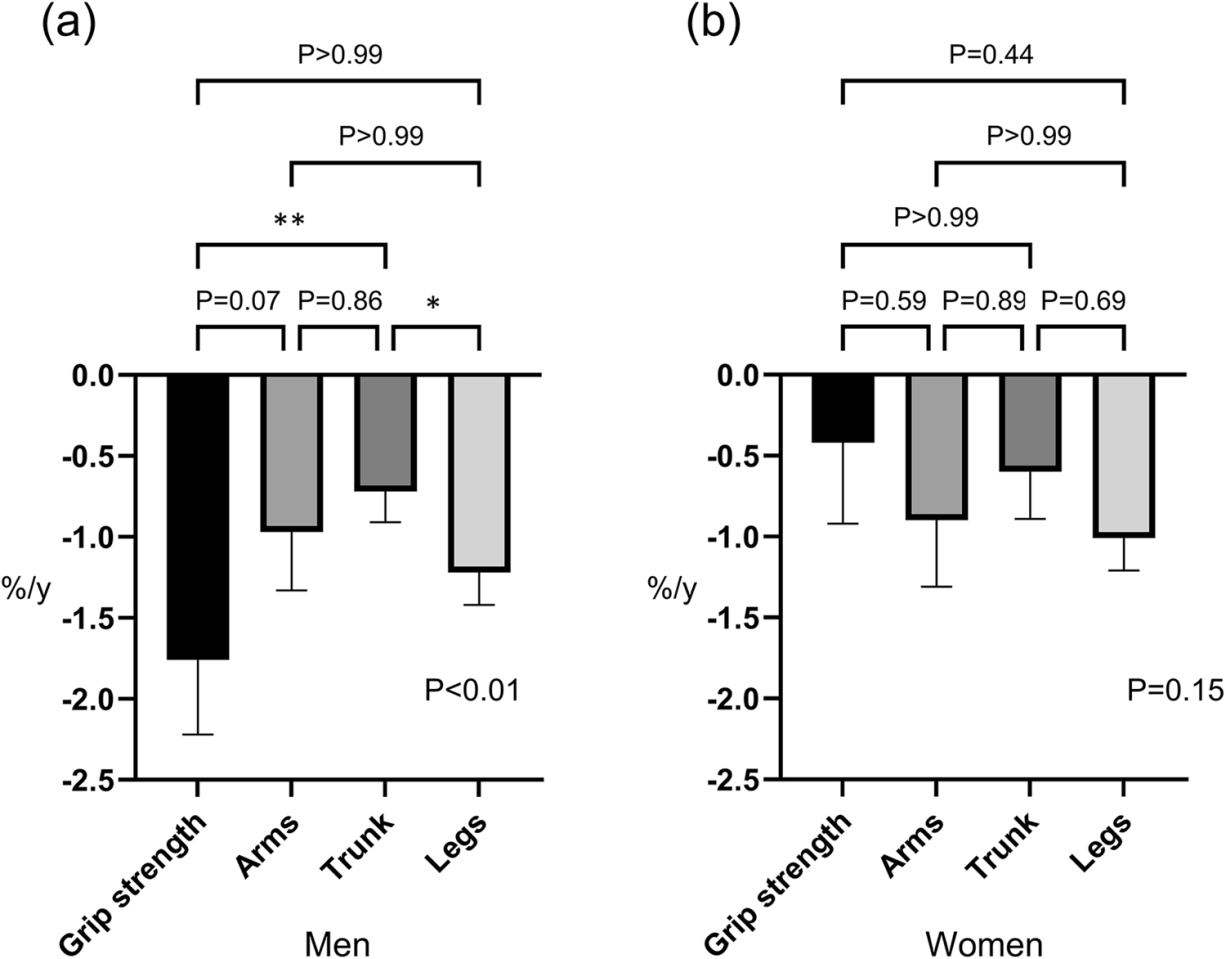
The annual rates of change in sarcopenia-relevant factors. *P < 0.05. **P < 0.01. The annual rates of change in grip strength and skeletal muscle of arms, trunk, and legs were − 1.76%, − 0.97%, − 0.72%, and − 1.22%/y for men (**a**) and − 0.42%, − 0.90%, − 0.60%, and − 1.01%/y for women (**b**), respectively.

In men, the grip strength decreased most markedly, and the rate of decrease in skeletal muscle mass was highest in the legs, followed by the arms and the trunk in order (*P* < 0.01) (Fig. 2). The rate of decline in grip strength and skeletal muscle mass of the legs was significantly greater than that of skeletal muscle mass of the trunk (*P* < 0.01). In women, there was a mild decrease in grip strength. As in men, the rate of decrease in the skeletal muscle mass in women was highest in the legs, followed by the arms and the trunk, but the differences were not statistically significant (*P* = 0.15).

### The comparison of the annual rate of change in sarcopenia-relevant factors

**Figure 3:**
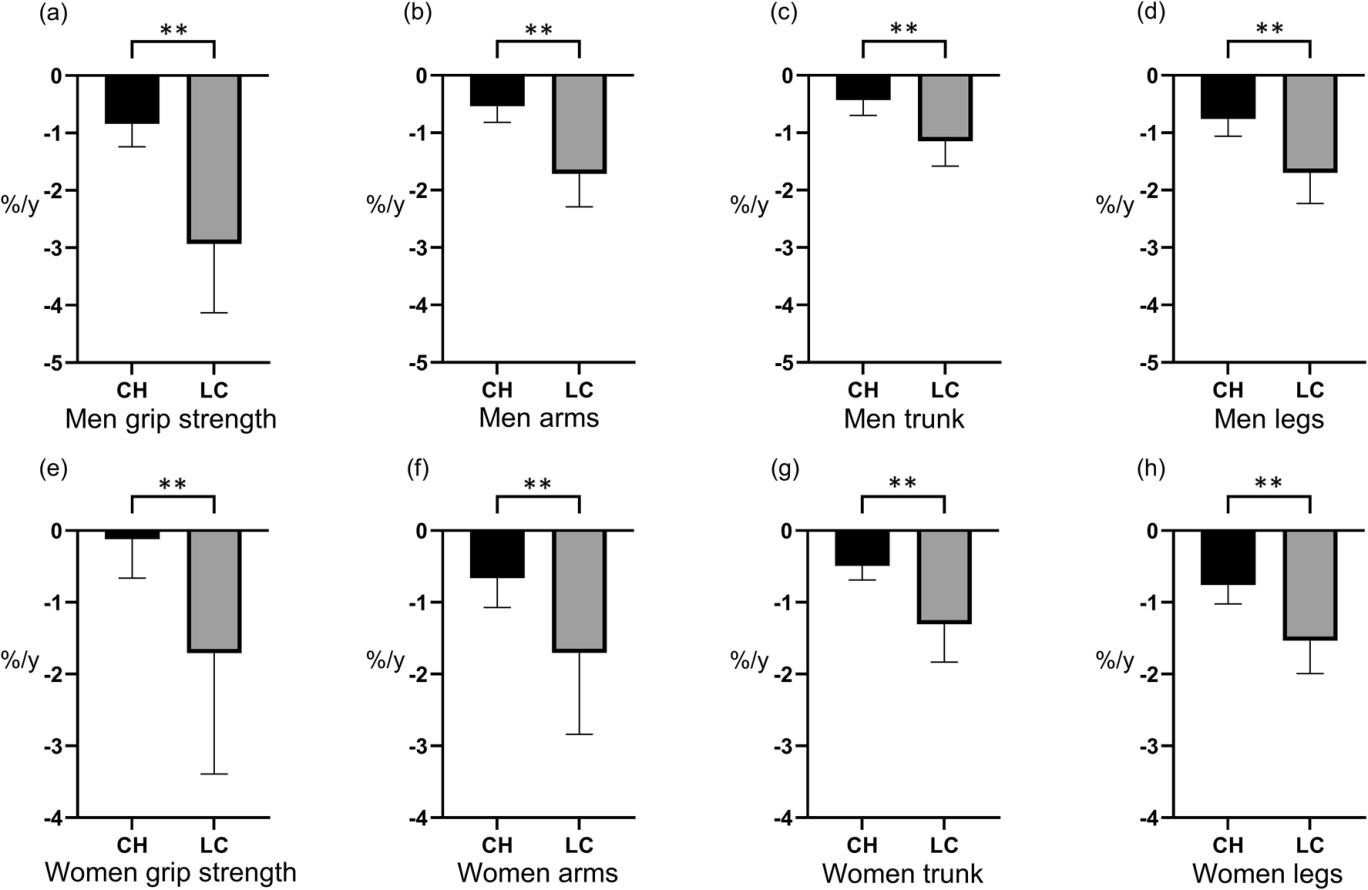
The comparison of the annual rate of change in sarcopenia-relevant factors between patients with chronic hepatitis (CH) and liver cirrhosis (LC). **P < 0.01. The annual rate of change in (**a**) grip strength and skeletal muscle of (**b**) arms, (**c**) trunk, and (**d**) legs of CH and LC men were − 0.84% vs. − 2.93% (*P* < 0.01), − 0.54% vs. − 1.71% (*P* < 0.01), − 0.43% vs. − 1.02% (*P* < 0.01), and − 0.76% vs. − 1.70% (*P* < 0.01), respectively. The annual rate of change in (**e**) grip strength and skeletal muscle of (**f**) arms, (**g**) trunk, and (**h**) legs of CH and LC women were − 0.12% vs. − 1.71% (*P* < 0.01), − 0.66% vs. − 1.71% (*P* < 0.01), − 0.49% vs. − 1.37% (*P* < 0.01), and − 0.76% vs. − 1.54% (*P* < 0.01), respectively.

In both men and women, the declining rate in grip strength and skeletal muscle mass in each region was significantly greater in LC patients than in CH patients (All *P* < 0.01) (Fig. 3). The declining rate in grip strength in men (− 2.93%/y) and the rates in grip strength (− 1.71%/y) and skeletal muscle mass of the arms (− 1.71%/y) in women were most pronounced in patients with cirrhosis.

### The comparison of the annual rate of change in sarcopenia-relevant factors according to the mALBI grade

**Figure 4:**
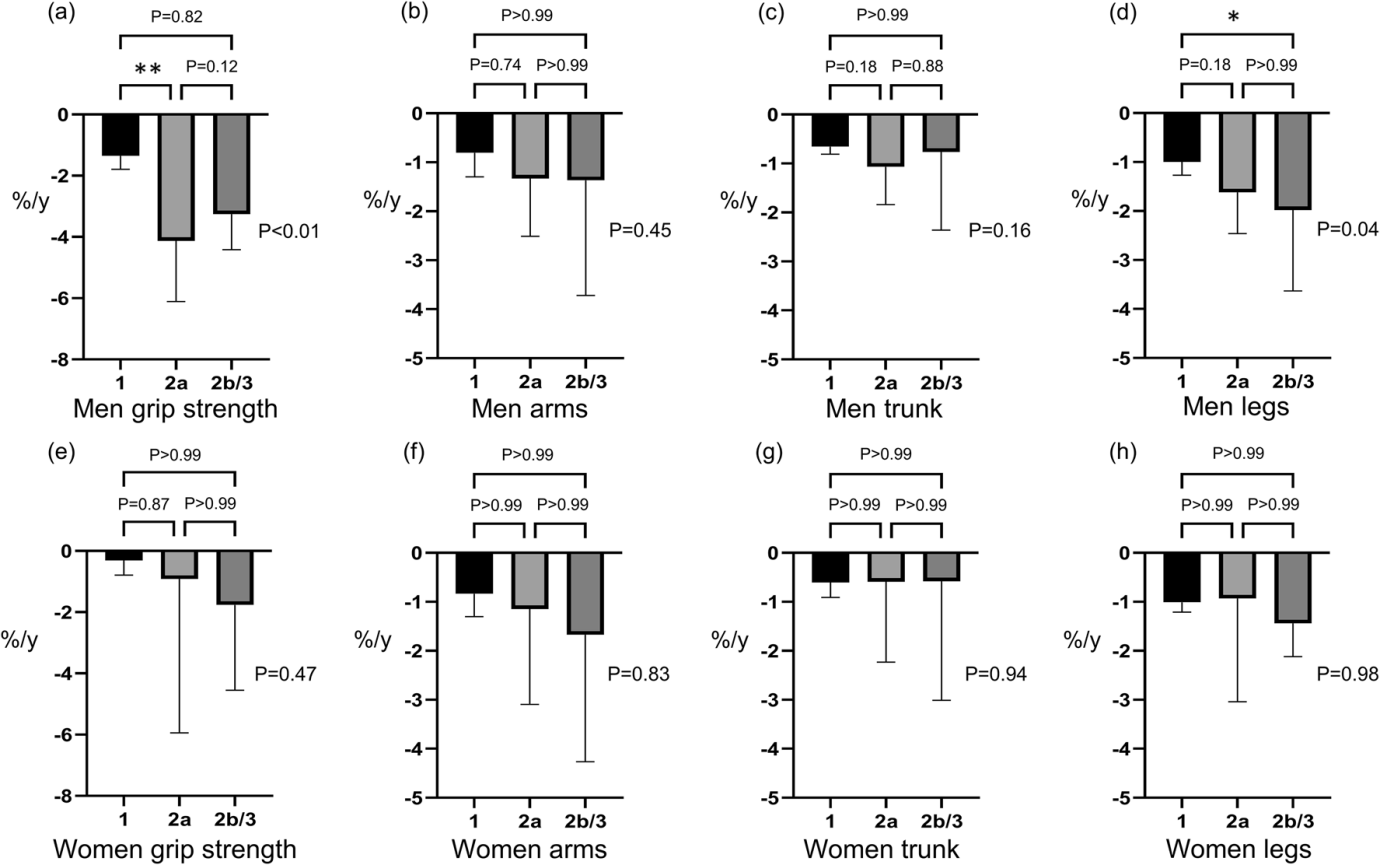
The comparison of the annual rate of change in sarcopenia-relevant factors according to the mALBI grade. *P < 0.05. **P < 0.01. The annual rates of change in (**a**) grip strength and skeletal muscle of (**b**) arms, (**c**) trunk, and (**d**) legs in mALBI 1, 2a, and 2b/3 men were − 1.35% vs. − 4.13% vs. − 3.26% (*P* < 0.01), − 0.80% vs. − 1.33% vs. − 1.37% (*P* = 0.45), − 0.66% vs. − 1.07% vs. − 0.76% (*P* = 0.16), and − 1.00% vs. − 1.62% vs. − 1.98% (*P* = 0.04), respectively. The annual rates of change in (**e**) grip strength and skeletal muscle of (**f**) arms, (**g**) trunk, and (**h**) legs in mALBI 1, 2a, and 2b/3 women were − 0.31% vs. − 0.92% vs. − 1.77% (*P* = 0.47), − 0.83% vs. − 1.15% vs. − 1.67% (*P* = 0.83), − 0.60% vs. − 0.59% vs. − 0.58% (*P* = 0.94), and − 1.01% vs. − 0.93% vs. − 1.44% (*P* = 0.98), respectively.

In men, patients with ALBI 2a had a significantly greater rate of decline in grip strength than patients with ALBI 1 (− 1.35%y vs. − 4.13%/y, *P* < 0.01), and patients with ALBI 2b/3 had a significantly greater rate of decline in the skeletal muscle mass of the legs than patients with ALBI 1 (− 1.00% vs. − 1.98%, *P* = 0.04) (Fig. 4). There was no significant difference in the rate of decline of sarcopenia-relevant factors among ALBI grades in women.

### Factors contributing to sarcopenia-relevant factors

A multiple regression analysis demonstrated that LC was independently associated with decreases in grip strength (*P* < 0.01) and skeletal muscle mass of the legs (*P* = 0.04) in men (Table 3). In men, age was independently associated with decreases in the skeletal muscle mass of the arms (*P* < 0.01) and trunk (*P* < 0.01) but not significantly associated with the change in grip strength (*P* = 0.53). In women, LC was an independent factor for both grip strength (*P* < 0.01) and skeletal muscle mass loss (Table 3). Age was independently associated with decreases in the skeletal muscle mass of the arms (*P* < 0.01) and trunk (*P* < 0.01) but not significantly associated with the decrease in grip strength (*P* = 0.10).

**Table 3:** Factors associated with the rate of change in sarcopenia-relevant factors.

	Grip strength		Arms		Trunk		Legs	
	B-value	*P*	B-value	*P*	B-value	*P*	B-value	*P*
*Men*								
Age (years)	− 0.02	0.53	− 0.07	< 0.01	− 0.06	< 0.01	− 0.02	0.18
Liver cirrhosis	− 2.95	< 0.01	− 1.17	0.09	− 1.07	0.22	− 1.35	0.04
Hepatocellular carcinoma	− 0.62	0.46	− 0.41	0.67	− 0.47	0.51	− 0.02	0.96
Diabetes mellitus	− 0.03	0.97	− 0.30	0.88	− 0.31	0.83	− 0.22	0.55
Etiology								
HBV		–		–		–		–
HCV	− 1.36	0.15	0.15	0.80	− 0.02	0.93	0.13	0.97
Alcohol	− 0.64	0.57	0.18	0.84	− 0.08	0.90	− 0.43	0.50
NAFLD	− 2.41	0.06	0.65	0.53	0.48	0.52	− 0.19	0.79
Others	− 2.83	0.15	− 0.12	0.91	− 0.97	0.79	− 1.14	0.10
Body mass index (kg/m^2^)	0.02	0.79	− 0.01	0.99	− 0.02	0.67	− 0.05	0.25
*Women*								
Age (years)	− 0.05	0.10	− 0.09	< 0.01	− 0.06	< 0.01	− 0.03	0.13
Liver cirrhosis	− 2.26	< 0.01	− 1.42	0.03	− 0.96	0.02	− 0.95	0.02
Hepatocellular carcinoma	0.60	0.53	− 0.81	0.41	− 0.69	0.27	0.62	0.32
Diabetes mellitus	− 0.50	0.58	− 0.06	0.91	− 0.06	0.88	0.68	0.08
Etiology								
HBV		–		–		–		–
HCV	− 1.76	0.13	− 0.19	0.86	− 0.01	0.95	0.01	0.99
Alcohol	2.09	0.46	− 2.98	0.11	− 2.21	0.08	− 0.19	0.86
NAFLD	− 2.74	0.13	0.65	0.59	0.38	0.61	1.15	0.12
Others	− 2.90	0.11	− 0.14	0.91	− 0.01	0.99	1.12	0.13
Body mass index (kg/m^2^)	− 0.07	0.47	0.04	0.54	0.01	0.88	− 0.01	0.87

HBV, hepatitis B virus; HCV, hepatitis C virus; NAFLD, nonalcoholic fatty liver disease.

## Discussion

Our study demonstrated that the progression of sarcopenia was greater in LC patients than in CH patients and that the decrease in grip strength was most prominent in the progression of sarcopenia in patients with LC.

The body composition and rate of decline in skeletal muscle mass differ among races^18,19^. The strength of the present study is that the population was limited to Asian subjects; thus, we were able to assess the pure effect of the muscle decline in patients with CLD, regardless of the effect of race. Most previous studies on similar topics have been cross-sectional^14–17,20^, therefore, another advantage of the present study is that it was not affected by baseline values, as we were able to serially examine the same study population. We were thus able to evaluate factors associated with the natural course of sarcopenia in patients with CLD.

The grip strength is a reliable and valid clinical tool commonly used to assess muscle strength in elderly people^2,3,20^. The advantages of grip strength include that it is simple, inexpensive, quick, repeatable and can be tested in a limited space. A recent worldwide cohort study reported that grip strength was a more significant predictor of all-cause and cardiovascular mortality than blood pressure^21^. This result suggests that measurement of grip strength may be useful in risk stratification for all-cause mortality. In the field of liver diseases, grip strength has also been proposed as a prognostic indicator in addition to hepatic functional reserve^22,23^. Recent studies have reported that the impact of skeletal muscle mass on the survival is limited compared to grip strength in patients with LC on the waiting list for liver transplantation and in HCC patients treated with systemic chemotherapy^23–27^.

We believe that our study was able to demonstrate why grip strength is more useful than skeletal muscle mass in predicting mortality. First, the decrease in grip strength according to the severity of liver disease was a greater than that in skeletal muscle mass. Indeed, the decrease in grip strength was three times or more greater in patients with LC than in patients with CH. Second, grip strength loss was the most prominent among the sarcopenia-relevant factors in patients with LC. Third, a multiple regression analysis demonstrated that LC was an independent factor associated with a decrease in grip strength, while age was associated with a decrease in skeletal muscle mass loss. This result suggests that grip strength is more directly affected by cirrhosis than skeletal muscle mass. Fourth, the skeletal muscle mass of the trunk was less likely to decrease than that of the extremities and grip strength, even in cirrhotic patients. The CT method measures the skeletal muscle mass of the trunk and is often used to assess the skeletal muscle mass in the field of liver disease. However, this method is reportedly less reflective of the prognosis than other modalities^9–13^. In contrast, the decline in grip strength was particularly pronounced in cirrhotic men in our study. Grip strength may thus have a significant impact on the overall prognosis, especially in male patients with LC. The European Working Group on Sarcopenia in Older People (EWGSOP) guidelines state that a low muscle strength is the primary parameter of sarcopenia, as muscle strength is the most reliable measure of the muscle function^2^. Furthermore, AWGS does not support the concept of pre-sarcopenia, which is a loss of skeletal muscle mass only, due to the lack of sufficient evidence regarding its prognostic value^3^. Our findings seem compatible with those statements, supporting the practical value of grip strength measurements in assessing sarcopenia.

The progression of sarcopenia was more pronounced in patients with LC than in patients with CH. In addition to age-related skeletal muscle loss, LC has unique multifactorial mechanisms, including protein-energy malnutrition, increased autophagy, ubiquitin proteasome, decreased serum testosterone, and growth hormone levels, hyperammonemia, physical inactivity, and increased myostatin levels, all of which lead to sarcopenia^28–30^. Indeed, the skeletal muscle loss rate increases with the progression of CLD. For patients with Child–Pugh class A, B, and C, the relative change in skeletal muscle volume per year has been reported to be − 1.3%, − 3.5%, and − 6.1%, respectively^31^. Regarding HCV-infected patients, skeletal muscle loss was more frequent in patients with LC than in patients with CH^32^. These results are consistent with the findings of this study.

Men showed a greater decline in grip strength than women in the present study. A cross-sectional national survey of independently dwelling elderly individuals in Japan reported the same results as in this study^20^. In both men and women, the rate of skeletal muscle mass decline was greatest in the legs, followed by the arms and then the trunk. Previous studies have found that the skeletal muscles mass of the legs shows a greater rate of decline than that of the arms, while the skeletal muscle mass of the trunk shows a less-marked decline with age in comparison to the skeletal muscles of the extremities^14,15^. Therefore, the progression of factors associated with sarcopenia in patients with CLD tends to be the same as in the elderly.

In addition to nutritional therapy, exercise therapy is recommended as a treatment for sarcopenia due to cirrhosis^33^. The results of this study may be helpful for determining the areas of resistance training on which to focus. For example, training of the legs, which are the most vulnerable to decline, may need to be emphasized. To this end, it is important to clarify the age at which the decline in skeletal muscle mass accelerates in patients with cirrhosis by body part, and further research is required.

Several limitations associated with the present study warrant mention. First, it was a single-center study with a relatively small sample size. In particular, the small number of patients with a poor liver function, such as mALBI 2a or 2b/3, may have contributed to the lack of clear stratification of the rate of change in sarcopenia-relevant factors. Second, we were unable to standardize the intervals for assessing sarcopenias. However, we believe the impact of this limitation is minimal, as we assessed most patients (70.2%) at intervals of 1–2 years and compared annualized changes. Third, there may have been some selection bias, as the prevalence of sarcopenia in this study was lower than that in ours and others’ similar studies^27,34^. The lower prevalence may be due to the exclusion of patients with advanced liver dysfunction for the initial or subsequent assessments. Fourth, the BIA method and grip strength measurements are associated with several limitations. The BIA method is known to overestimate skeletal muscle mass in the presence of edema and ascites, which are frequently seen in patients with cirrhosis^35^. Cases with obvious edema or ascites were excluded from this study, but mild cases may have been included. Although grip strength is the most commonly used index of muscle strength, this study did not assess other indices of muscle strength and physical ability besides the grip strength (e.g., walking speed or chair stand test). Past studies have shown that muscle weakness varies by sex, with men showing a significant decline in grip strength and women showing a similar decline in walking speed, which may affect the results^20^.

In conclusion, the progression of skeletal muscle mass and grip strength decline were more severe in LC patients than in CH patients. Grip strength loss was most prominent in the progression of sarcopenia in patients with LC.

## Materials and methods

### Patient selection and study design

This was a single-center, observational study based on data collected from a university hospital. We analyzed patients with CLD who underwent two tests for sarcopenia using a BIA method with intervals of at least one year between 2016 and 2021. If the patient had been tested more than three times, the results of the first and second tests were used.

The inclusion criteria for patients were as follows: (1) a normal cognitive function and (2) independence in activities of daily life (ADL). The exclusion criteria were as follows: (1) visible edema or massive ascites, (2) severe renal, cardiopulmonary, or musculoskeletal disorders, and cerebrovascular disease, (3) malignant tumor except for hepatocellular carcinoma (HCC), and (4) advanced-stage (Barcelona Clinic Liver Cancer classification C or D) HCC^36^. In addition, patients with edema or ascites were excluded because the BIA method may overestimate skeletal muscle mass^37^.

The opt-out approach was used to obtain informed consent from all patients prior to the study. The study complied with the provisions of the 1964 Declaration of Helsinki and was approved by the Ethics Committee of our hospital (MH2019-133).

### Assessment of body composition and hand grip strength evaluations

We used an InBody720 device (Biospace, Seoul, Korea) to perform a BIA for evaluating the skeletal muscle mass. The assessment of skeletal muscle mass by the BIA method is used in the Japan Society of Hepatology diagnostic criteria for sarcopenia for patients with CLD as well as in the AWGS criteria for diagnosing sarcopenia in the elderly^3,9^. The InBody 720 adopt a tetrapolar, eight-point tactile electrode system that separately evaluates the impedance of the arms, trunk, and legs. Patients were instructed to stand on the scale holding a handrail with attached metal grip electrodes. The patients then extended their arms at an abduction angle of about 20° to the side. The InBody 720 automatically measured the weight, total fat mass, and skeletal muscle mass of the arms, trunk, and legs. The skeletal muscle index (SMI) was normalized by dividing the total appendicular skeletal muscle mass (arms and legs) by the square of the height. The skeletal muscle mass of the arms, trunk and legs was also normalized by dividing by the square of height and expressed as the SMI of the arms, trunk and legs. The grip strength was measured using a Smedley-type digital hand dynamometer (T.K.K.5401; Takei Scientific Instruments, Niigata, Japan) with the elbow straight in the standing position. The maximum strength over two trials for each hand was averaged for the analysis. This study defined sarcopenia-relevant factors as the grip strength and skeletal muscle mass of the arms, trunk, and legs.

The annual rate of change (%/y) each in the grip strength and the skeletal muscle mass was calculated using the following equation: (second measurement − first measurement)/first measurement × 100/observation period (year).

To clarify the effect of cirrhosis on the development of sarcopenia-relevant factors in CLD patients, each parameter was compared between patients with chronic hepatitis (CH) and LC. To determine the effect of the hepatic reserve on the development of sarcopenia-relevant factors in CLD patients, we compared each parameter by the modified albumin-bilirubin (mALBI) grade, which was calculated as previously reported^38^.

### The diagnosis of sarcopenia

Sarcopenia was defined according to the AWGS definition, which included both a low muscle mass and strength^3^. A low muscle mass was defined as an SMI < 7.0 and < 5.7 kg/m^2^ in men and women, respectively. A low muscle strength was defined as a grip strength < 28 in men and < 18 kg in women.

### Clinical and laboratory assessments

All measurements were carried out in the morning after an overnight fast of at least 10 h. Clinical characteristics and laboratory data were collected on the day of the sarcopenia assessment. The body mass index (BMI) was calculated by dividing the weight by the square of the height (kg/m^2^). CH and LC were diagnosed comprehensively based on clinical and laboratory findings (e.g. platelet count under 10 × 10^4^ cells/μL or elevated fibrosis markers of the liver), definite imaging findings of cirrhosis, esophageal varices, collateral blood vessels, and liver biopsy findings^39,40^.

### Statistical analyses

Continuous variables are expressed as the mean and standard deviation when normally distributed and as the median and range when not normally distributed. Categorical variables are expressed as the number of patients and percentages (%). We used the Mann–Whitney U-test to analyze continuous variables and Fisher’s exact test to analyze categorical variables. Multiple comparisons were performed using the Kruskal–Wallis test with Bonferroni multiple comparisons. A multiple regression analysis was used to identify the independent predictors of the rate of change in sarcopenia-relevant factors. All tests were 2-sided, and *P* values of < 0.05 were considered to indicate statistical significance in all analyses. All statistical analyses were performed using the GraphPad Prism software program (version 9; GraphPad Software, San Diego, CA, USA).

## Data Availability

The data that support the findings of this study are available from the corresponding author upon reasonable request.
